# DNA accelerates the protease inhibition of a bacterial serpin chloropin

**DOI:** 10.3389/fmolb.2023.1157186

**Published:** 2023-03-29

**Authors:** Jiawei Xu, Wei Ye, Ting Ting Yang, Teng Yan, Haiyan Cai, Aiwu Zhou, Yufeng Yang

**Affiliations:** ^1^ Department of Bioengineering, Zunyi Medical University Zhuhai Campus, Zhuhai, Guangdong, China; ^2^ Department of Preventive Dentistry, The Ninth People’s Hospital, Shanghai Jiao Tong University School of Medicine, Shanghai, China; ^3^ Department of Pathophysiology, Shanghai Jiao Tong University School of Medicine, Shanghai, China

**Keywords:** prokaryotic serpins, crystal structure, thrombin, heparin, DNA, template mechanism

## Abstract

Serine protease inhibitors (Serpins) are the most widely distributed protease inhibitors in nature and have been identified from all kingdoms of life. Eukaryotic serpins are most abundant with their activities often subject to modulation by cofactors; however, little is known about the regulation of prokaryotic serpins. To address this, here we prepared a recombinant bacteria serpin, termed chloropin, derived from green sulfur bacteria Chlorobium limicola and solved its crystal structure at 2.2 Å resolution. This showed a canonical inhibitory serpin conformation of native chloropin with a surface-exposed reactive loop and a large central beta-sheet. Enzyme activity analysis showed that chloropin could inhibit multiple proteases, such as thrombin and KLK7 with second order inhibition rate constants at 2.5×10^4^ M^−1^s^−1^ and 4.5×10^4^ M^−1^s^−1^ respectively, consistent with its P1 arginine residue. Heparin could accelerate the thrombin inhibition by ∼17-fold with a bell-shaped dose-dependent curve as seen with heparin-mediated thrombin inhibition by antithrombin. Interestingly, supercoiled DNA could accelerate the inhibition of thrombin by chloropin by 74-fold, while linear DNA accelerated the reaction by 142-fold through a heparin-like template mechanism. In contrast, DNA did not affect the inhibition of thrombin by antithrombin. These results indicate that DNA is likely a natural modulator of chloropin protecting the cell from endogenous or exogenous environmental proteases, and prokaryotic serpins have diverged during evolution to use different surface subsites for activity modulation.

## 1 Introduction

Serine protease inhibitors (Serpins) are the largest and most widely distributed family of protease inhibitors found in animals, plants, bacteria, viruses, and archaea (Huber and Carrell, 1989; Gettins, 2002; Irving et al., 2002; Heit et al., 2013; Spence et al., 2021). Serpins are generally composed of 330–500 amino acid residues with similar secondary and tertiary structures. It is folded into a metastable conformation (also called the stressed state) consisting of eight to nine α-helices (A-I), 3 β-sheets (A, B, C), and a surface exposed reaction center loop (RCL). The sequence diversity of the RCL is often the key determinant of the specificity of serpins. The inhibitory serpins do not inhibit their target proteases by the typical lock-and-key mechanism of small protease inhibitors such as Kunitz-type inhibitors. Instead, serpins use an unusual S-to-R conformational change to inhibit target serine proteases (Huber and Carrell, 1989; Gettins, 2002).

Serine proteases catalyze the peptide bond cleavage through a two-step process (Gettins, 2002). Initially, the catalytic serine of the active site triad performs a nucleophilic attack on the peptide bond of the substrate. This releases the new N-terminus and forms a covalent ester bond between the enzyme and the substrate. This covalent complex between enzyme and substrate is called an acyl-enzyme intermediate. For standard substrates, the ester bond is hydrolyzed and the new C-terminus is released to complete catalysis. However, when the RCL of a serpin is cleaved by a protease, the serpin rapidly undergoes a dramatic conformational change where the cleaved RCL is swiftly inserted into the serpin’s central beta-sheet, in which the metastable conformation (stressed state) of native serpin is converted to a hyper stable conformation (the relaxed state). This conformation switch is often called the S-to-R transition. The protease covalently linked to RCL by an ester bond in the acyl-enzyme intermediate stage is translocated about 70Å to the other side of the serpin moleculebuchon (Stratikos and Gettins, 1997; Huntington et al., 2000; Gettins, 2002). This leads to the disruption of the protease’s catalytic site (Stratikos and Gettins, 1997; Huntington et al., 2000). The distorted protease can only hydrolyze the acyl-enzyme intermediate extremely slowly and so the protease remains covalently attached for days to weeks. Serpins are classed as irreversible inhibitors and as suicide inhibitors since each serpin protein permanently inactivates a single protease, and can only function once (Huntington et al., 2000; Gettins, 2002).

The structure, function, and regulatory mechanisms of serpins in eukaryotes have been meticulously studied over the last few decades (Huber and Carrell, 1989; Gettins, 2002; Irving et al., 2002; Heit et al., 2013; Spence et al., 2021). Inhibitory eukaryotic serpins participate in regulating many physiological processes such as blood coagulation (Ikezoe, 2021), inflammatory responses (Greene et al., 2016), tissue remodeling (François et al., 2014), and apoptosis (Maas and de Maat, 2021), while non-inhibitory eukaryotic serpins have evolved to a variety of other functions, such as hormone transporters (Zhou et al., 2006; Klieber et al., 2007), tumor suppressor (Zhang et al., 1997), and molecular chaperones (Widmer et al., 2012), etc. Deficiencies in these eukaryotic serpins are associated with many diseases (Hunt and Dayhoff, 1980; Lomas et al., 1992; Gettins, 2002). Notably many of these eukaryotic serpins have evolved to have auxiliary subsites (for example, extra surface loops, extended N or C-terminal tails) (Gettins and Olson, 2009) or adopted cofactors such as heparin, protein Z, phospholipids, calcium, and vitronectin, etc. for the modulation of their functions (Wei et al., 2009; Huang et al., 2011; Huang et al., 2012; Huang et al., 2022). These cofactors could accelerate the interaction between a serpin and its target protease through either an allosteric or template mechanism. For example, the unique heparin pentasaccharide could specifically bind to antithrombin near its helix D and allosterically induce a conformation change in the central beta-sheet and the RCL of antithrombin leading to accelerated inhibition of activated factor X (Jin et al., 1997). Whereas heparin promotes the inhibition of thrombin by antithrombin through bridging antithrombin and thrombin together and in a bell-shaped dose-dependent manner (Dementiev et al., 2004; Li et al., 2004). At lower concentrations, one cofactor molecule could bridge serpin and protease simultaneously and accelerate the rate of inhibition, however, at the higher concentration of cofactors, antithrombin and thrombin bind two different cofactors, and the overall rate of inhibition decreases. Protein Z could bind protein Z-dependent inhibitors and promote the inhibition of activated factor X on the phospholipid membrane in the presence of calcium using a similar template mechanism (Han et al., 1999; O'Keeffe et al., 2004; Huang et al., 2011; Huang et al., 2012).

In contrast, there are only limited studies on prokaryotic serpins (Zhang et al., 2007; Mkaouar et al., 2021). In 2002, Irving et al. (Irving et al., 2002) identified for the first time the gene sequences of 12 serpins in the genomes of bacteria and archaea, extending the serpin family to all branches of life. Subsequently, Tk-Serpin, derived from the extremely thermophilic bacterium Thermococcus kodakaraensis, was found to be acid-tolerant and heat-stable, capable of inhibiting proteases in the environment and thus protecting the bacterium itself (Tanaka et al., 2011). The discovery of miropin (Ksiazek et al., 2015; Goulas et al., 2017), the first human periodontitis-associated serpins with broad-spectrum inhibition, has extended the study of prokaryotic serpins into the realm of human parasitic bacteria. The other prokaryotic serpin siropin (Mkaouar et al., 2016) encoded by Eubacterium sireaum significantly induced recovery in mice with enteritis and is considered a good candidate for inhibition of inflammation-associated proteases. Recently comprehensive analysis of a 6,000-sequence phylogeny including serpins from all kingdoms of life has identified many new prokaryotic serpins. Detailed structural and functional studies of these prokaryotic serpins may shed light on the origin and evolution of the serpin superfamily.

For this purpose, our laboratory has selected about 20 new prokaryotic serpins for detailed structural analysis and one of them is termed chloropin as it is derived from green sulfur bacteria Chlorobium limicola, which is an anaerobic green phototrophic bacterium. It can reduce sulphides to fix carbon dioxide in the presence of light and plays an important role in the anaerobic cycling of materials in aquatic ecosystems (Verté et al., 2002). Here we showed that chloropin is an inhibitory serpin and its activity could be regulated by cofactors such as heparin and DNA and DNA is likely a natural cofactor for chloropin.

## 2 Materials and methods

### 2.1 Materials

The cDNA of chloropin (UniProt B3ECZ8) derived from Chlorobium limicola was synthesized by GENEWIZ (Nanjing, China). *Escherichia coli* BL21 (DE3) was purchased from Tiangen. Columns for protein purification were purchased from GE Healthcare. SDS-PAGE precast gels and MOPS Running Buffer were purchased from Genscript. Proteases, including human neutrophil elastase (HNE), pancreatic elastase, trypsin, kallikrein 1 (KLK1), kallikrein 7 (KLK7), proteinase 3 (PR3), thrombin, activated factor IX (FIXa), activated factor X (FXa), activated factor XI (FXIa), activated protein C (APC), tissue-type plasminogen activator (tPA), cathepsin B and cathepsin G, were purchased from Haematologic Technologies. The fluorescence-quenched KLK7 substrate Abz-Asn-Leu-Tyr-Arg-Val-Glu-Gln-Lys (Dnp) was synthesized from BankPeptide, while the thrombin substrate S-2238 was purchased from Chromogenix. Low molecular weight heparin (LMWH) with an average molecular weight of approximately 5 kDa was purchased from Sigma. All crystallization reagents and kits were purchased from Hampton Research.

### 2.2 Cloning, mutation, expression, and purification of recombinant proteins

The chloropin gene sequence at residues 54-420 was cloned in the *E. coli* expression vector pSumo3 where the Sumo3 tag was fused with chloropin. All chloropin mutants were constructed by inverse PCR mutagenesis using the KOD Plus kit (TOYOBO). The expression plasmid was then transformed into BL21 (DE3) and individual colonies were picked and incubated in a 2YT medium at 37°C until the optical density of the broth at 600 nm was 0.6. At this point, the temperature was lowered to 20°C and isopropyl-β-d-1-thiogalactoside (IPTG) was added at a final concentration of 0.1 mM with culture incubated for further 16 h. Cells were collected by centrifugation and resuspended in buffer (20 mM Tris-HCl, pH 7.4, 0.5 M NaCl, and 20 mM imidazole) and lysed at 600 bar pressure. The supernatant of the cell lysate was loaded onto a HisTrap column and the bound protein was eluted with a linear gradient of 20–300 mM imidazole. The fractions containing the fusion protein were digested with protease SENP2 and then loaded onto a new HisTrap column to remove the released His-SUMO3 tag. Recombinant chloropin was further purified by a HiTrap SP column and a gel filtration column (HiLoad 16/600 Superdex 75 pg). It appeared that sometimes chloropin is susceptible to certain proteolysis where two protein bands could be seen on an SDS gel from the sample after long-term storage.

### 2.3 Crystallization, data collection, and structure elucidation

Initial crystal screening was performed with a 200 nL solution of homogeneous chloropin (8 mg/mL) mixed with 200 nL of Hampton Research crystallization kit reagents in MRC2 crystallization plates at 20°C using the sitting-drop vapor phase diffusion method. After initial hits, the crystals were grown from a solution containing 25% PEG MME 550, 0.1 M MES pH 6.5, and 0.01 M Zinc Sulfate. Single crystals were retrieved with Cryoloop and placed in cryoprotectant containing 25% glycerol. The X-ray diffraction was collected at SSRF 18U (Shanghai, China) with data integrated using iMosflm and scaled using Aimless in the CCP4i software package (Collaborative Computational Project, 1994). Comserpin (PDB 6EE5) (Verté et al., 2002), which shares ∼42% sequence identity with chloropin, was then used as the search model for molecular replacement in Phaser (McCoy et al., 2005). The structure of chloropin was refined with Refmac5 (Winn et al., 2001) and the model was built with Coot software (Emsley and Cowtan, 2004). The surface electrostatic representation was analyzed by APBS (Jurrus et al., 2018). The figures were created using the open-source program PyMOL. The detailed crystallographic statistics are shown in Table 1 and the structure factors and coordinates have been deposited in the protein data bank with accession number 8GXV.

**TABLE 1:** Data processing, refinement, and model statistics.

Crystal	Chloropin (PDB 8GXV)
Data collection	
Beamline	SSRF 18U
Space group	P2_1_ 2 2_1_
Cell dimensions	
α, β, γ (°)	90, 90, 90
a, b, c (Å)	60.66, 60.91, 130.16
Wavelength (Å)	0.9792
Resolution (Å)	60.91-2.20 (2.279-2.20)
Total NO. of observation	50408 (4908)
Total NO. unique	25206 (2453)
Rmerge (%)	0.0297 (0.1342)
I/σI	15.11 (4.44)
Completeness (%)	99.92 (99.96)
Multiplicity	2.0 (2.0)
Refinement	
Resolutions (Å)	60.91–2.20
NO. of reflections/free	25194/1234
NO. of residues	353
NO. of atoms	2852
Rwork/Rfree	0.201/0.247
B-factors (Å2)	30.48
RMSD	
Bond lengths (Å)	0.005
Bond angles (°)	0.84
Ramachandran plot	
In Preferred Region (%)	97.71
In Allowed Region	2.29
Rotamer outliers (%)	0.00

### 2.4 Proteases inhibition, stoichiometry inhibition, and the 2nd-order association rates

To assess the inhibitory activity of chloropin towards various serine proteases, chloropin (3 μg) was mixed with 0.4 μg of 14 different proteases (human neutrophil elastase, pancreatic elastase, trypsin, kallikrein 1, kallikrein 7, proteinase 3, thrombin, activated factor IX, activated factor X, activated factor XI, activated protein C, tissue-type plasminogen activator, cathepsin B, and cathepsin G) respectively, and incubated at 37°C for 30 min. The mixtures were then analyzed by SDS-PAGE. The covalently linked serpin-protease complexes would migrate slower than the serpin and protease and this provided a preliminary indication of the inhibitory effect of chloropin on proteases. In addition, 10 μM chloropin was mixed with 20 μM proteases (KLK7 and thrombin) and reacted at 37°C for 2 h. The molecular weight of the C-terminal peptide of chloropin was then assayed by mass spectrometry (MS) to infer the protease cleavage site on the reactive center loop of chloropin (Ksiazek et al., 2015).

The stoichiometry of inhibition (SI) was measured by incubating 0.25 μM thrombin or KLK7 with increasing concentrations of chloropin (0–2 μM) for 12 h at room temperature. Residual protease activity was determined by diluting the reaction mixture into 0.1 mM chromogenic thrombin substrate S-2238 (Chromogenix) or fluorescence-quenched KLK7 substrate Abz-Asn-Leu-Tyr-Arg-Val-Glu-Gln-Lys (Dnp) (Maggiora et al., 1992). When the KLK7 substrate is intact, the signal emitted by Ortho-aminobenzoic acid (Abz) is absorbed by 2,4-dinitrophenyl (Dnp). However, when the substrate is hydrolyzed and Abz is released, the signal can be collected at 420 nm with photoexcitation at 320 nm. The remaining protease activity was measured from the changes of absorbance at 405 nm for thrombin or from the changes of fluorescence at 420 nm for KLK7. The plot of the ratio of serpin/protease against the residual protease activity yielded the SI values for the inhibition as described previously (Wei et al., 2016). Each measurement was repeated three times with the mean value ±SD calculated.

The second-order association rates of protease inhibition by chloropin were determined under pseudo-first-order conditions. Thrombin or KLK7 (10 μL of 100 nM) was mixed with 10 μL of 2 μM chloropin in the absence or the presence of cofactors for different time intervals. The residual protease activities were determined by diluting the reaction mixture into the assay buffer (PBS 0.1% PEG8000 0.1 mg/mL BSA) containing 0.1 mM corresponding substrates. The observed rate constant, *k*_obs_, was obtained from the slope of a semi-log plot of the residual protease activity against time. The apparent second-order rate constant, *k*_app_, was calculated by linear fitting of *k*_obs_versus the initial inhibitor concentration.

The effect of low molecular weight heparin (LMWH) on the interactions between chloropin and thrombin was similarly assessed as previously described (Wei et al., 2016; Ma et al., 2020). A total of 1 µM chloropin was incubated with different concentrations of LMWH (0, 0.005, 0.025, 0.05, 0.25, 0.5, 1, and 2.5 mg/mL) for 5 min at room temperature, and then mixed with 100 nM thrombin and the residual thrombin activity was measured using 0.2 mM S-2238 at different time intervals. The observed rate constants, *k*_obs_, were then plotted with the concentrations of LMWH. The effect of supercoil and linear DNA derived from a 6kb plasmid on the thrombin inhibition by chloropin was similarly probed where 375 nM chloropin was incubated with 75 nM thrombin in the presence of 0–25.6 nM plasmid DNA (6,000 bp).

### 2.5 Heparin affinity chromatography of chloropin variants

The relative heparin binding affinities of recombinant chloropin variants were assessed by a heparin-Sepharose column (Wei et al., 2016; Ma et al., 2020). About 500 µg of protein was loaded onto a 1 mL HiTrap heparin column pre-equilibrated with 10 mM Tris-HCl, pH 7.4, 1mM EDTA, and eluted with a 20-column volume of 100–500 mM NaCl gradient. The protein absorbance at 280 nm was recorded with the salt concentration of peaks identified and the fractions analyzed by SDS-PAGE.

### 2.6 Model of the chloropin-thrombin-DNA ternary complex

There are about a dozen different serpin-proteases Michaelis complex crystal structures in the Protein Data Bank with the relative positions of serpin and protease somewhat different. Due to the lack of crystal structures of DNA complexed with either thrombin or chloropin, the model of thrombin and chloropin Michaelis complex was prepared from the thrombin/antithrombin complex structure (PDB 1SR5) (Dementiev et al., 2004) with chloropin superposed onto antithrombin. This resulted in minimum clashes between the two molecules. The binding mode of DNA on chloropin was first evaluated with the online ZDOCK server (Pierce et al., 2011) and then one doublestranded DNA molecule was tentatively extended to bridge both the positively charged surface areas of thrombin and helix D of chloropin. This showed the relative positions of the components in the chloropin-thrombin-DNA ternary complex.

## 3 Results

### 3.1 Preparation and structural characterization of recombinant chloropin

**FIGURE 1:**
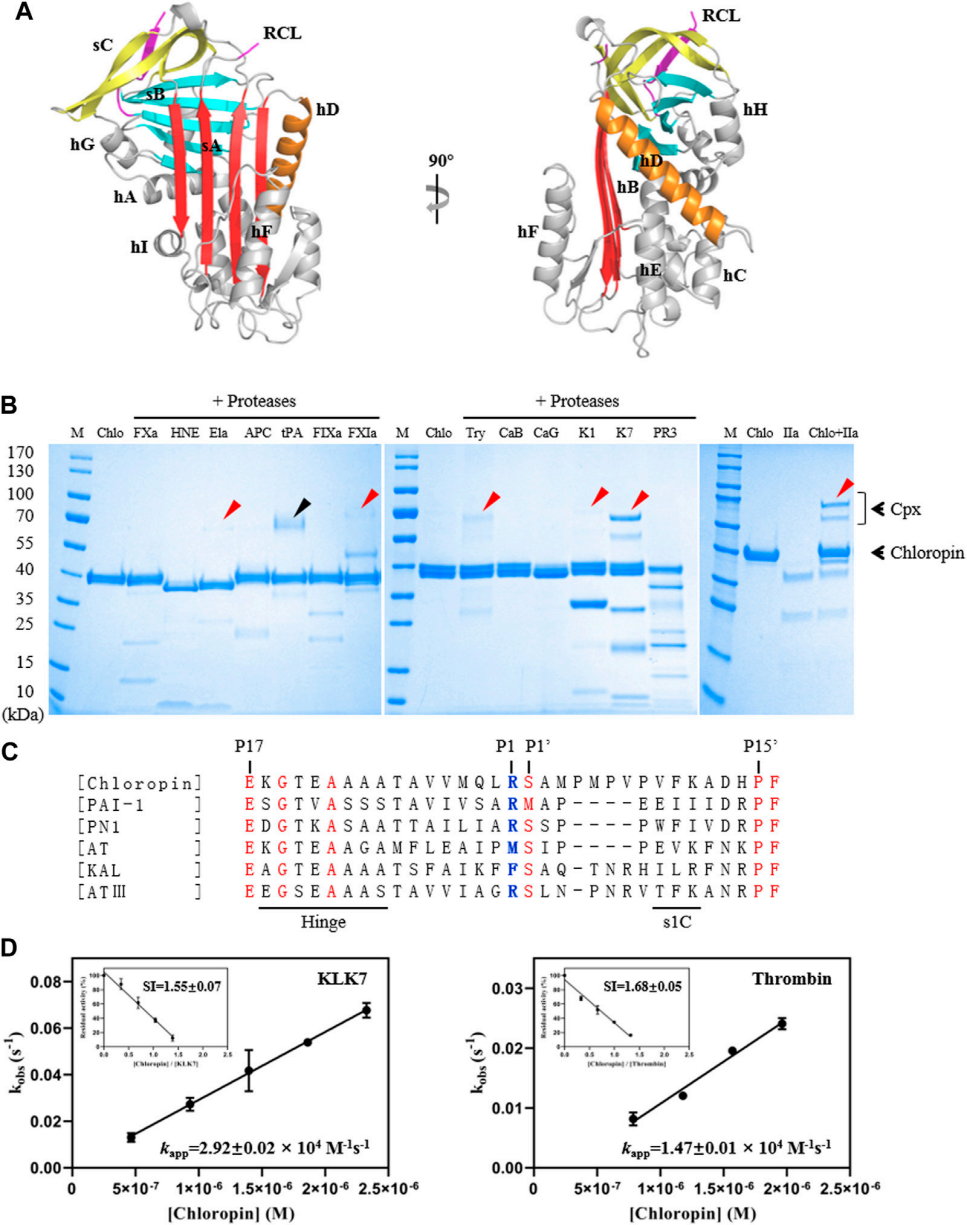
Structure and activity analysis of chloropin. **(A)** The crystal structure of chloropin contains 9 α-helices (hA-I), 3 β-sheets (A–C), and a reaction center loop (RCL). Part of the RCL was not resolved in the structure due to its flexibility which is a common feature in many serpins. **(B)** 3 μg chloropin was incubated with 0.4 μg proteases including activated factor X (FXa), human neutrophil elastase (HNE), pancreatic elastase (Ela), activated protein C (APC), tissue-type plasminogen activator (tPA), activated factor IX (FIXa), activated factor XI (FXIa), trypsin (Try), cathepsin B (CaB) cathepsin G (CaG), kallikrein 1 (K1), kallikrein 7 (K7), proteinase 3 (PR3) and thrombin (IIa) respectively at 37°C for 30 min and the complex formation was then analyzed by SDS-PAGE. The red arrows are used to indicate the chloropin-protenase covalent complexes (Cpx), and the ∼70 kDa band in the tPA lane is the protease itself (marked by a black arrow). **(C)** Alignment of RCL sequences of chloropin (Uniprot B3ECZ8) with other heparin-binding inhibitory serpins such as PAI-1 (Uniprot P05121), protease nexin 1 (Uniprot P07093), antitrypsin (Uniprot A0A024R6I7), kallistatin (Uniprot P29622) and antithrombin (Uniprot P05154). The conserved amino acids are shown in red and the active center P1 is shown in blue. **(D)** Determination of the stoichiometry of inhibition (SI) and association rate constants (*k*_app_) of chloropin’s inhibition of KLK7 (left panel) and thrombin (right panel).

Recombinant chloropin protein was expressed in *E coli* with most of the fusion protein as insoluble inclusion body. Nevertheless, about 5 mg chloropin could be purified from the supernatant of the cell lysate from 1-L cell culture by Histrap and Hitrap-SP columns. The gel filtration chromatography and SDS-PAGE analysis showed that the prepared chloropin was homogenous and in a monomeric state. To characterize the structural features of chloropin, we crystallized recombinant chloropin and determined its crystal structure at 2.2 Å resolution (Table 1). There was one copy of chloropin in the asymmetric unit, which showed a canonic inhibitory native serpin conformation of 9 α-helices wrapping 3 β-sheets similar to that of other prokaryotic serpins (Figure 1A) (Fulton et al., 2005; Zhang et al., 2007; Goulas et al., 2017). Chloropin has a large central β-sheet A and a surface-exposed active center loop, which is largely invisible due to high flexibility. The central β-sheet A on the front of the structure consists of 5 strands of which s3A and s5A are parallel. During the protease inhibition process, the reactive loop of the serpin is expected to be inserted into the central β sheet as a middle strand. Sequence analysis showed that chloropin has a typical reactive loop of inhibitory serpins with Arg-Ser at the P1-P1′ and smaller residues at the hinge region which are largely conserved amongst known inhibitory serpins (P17–P9: ExGTEAAAA, x: E/K/R) (Gettins, 2002; Spence et al., 2021) (Figure 1C). Overall, these structural features of chloropin indicate that it is likely an inhibitory serpin.

### 3.2 Specificity of chloropin towards serine proteases

To test the activity and specificity of chloropin, thirteen commonly known serine proteases were selected and incubated with chloropin and the covalently linked serpin-protease complex formation was assessed by SDS-PAGE. As shown in Figure 1B, bands corresponding to the serpin-protease complexes could be observed from the incubations of chloropin with elastase, FXIa, trypsin, KLK1, KLK7, and thrombin on the gel, indicating that chloropin could inhibit these serine proteases, while the chloropin bands shifted downwards when incubated with HNE, cathepsin G and proteinase 3 indicating chloropin is likely a substrate of these proteases with cleavage occurred at multiple sites. As significant more complexes were observed from the KLK7 and thrombin incubations, we then performed kinetic studies on their interactions with chloropin. The SI value for inhibition of KLK7 by chloropin was 1.55 and *k*_app_ was 2.92×10^4^ M^−1^s^−1^, whereas the SI for thrombin inhibition was 1.68 and *k*_app_ was 1.47×10^4^ M^−1^s^−1^ (Figure 1D). The derived second-order inhibition rate constants (*k*_2_) of chloropin against KLK7 and thrombin were 4.5×10^4^ M^−1^s^−1^ and 2.5×10^4^ M^−1^s^−1^ respectively. These values are close to those of the other bacterial serpins such as miropin (Ksiazek et al., 2015). Furthermore, the chloropin-protease complexes were analyzed by mass spectrometry to assess the molecular weight of C-terminal peptide of choloropin released by protease cleavage. This showed that a peptide of 4146.5Da could be detected from both chloropin-thrombin and chloropin-KLK7 complexes. This is consistent with the predicted P1 residue Arg383 of chloropin (Supplementary Figure S1). Overall, these results showed that chloropin is an inhibitory serpin and could preferentially inhibit serine proteases such as KLK7 and thrombin using Arg383 as the P1 residue.

### 3.3 Heparin promotes thrombin inhibition by chloropin

**FIGURE 2:**
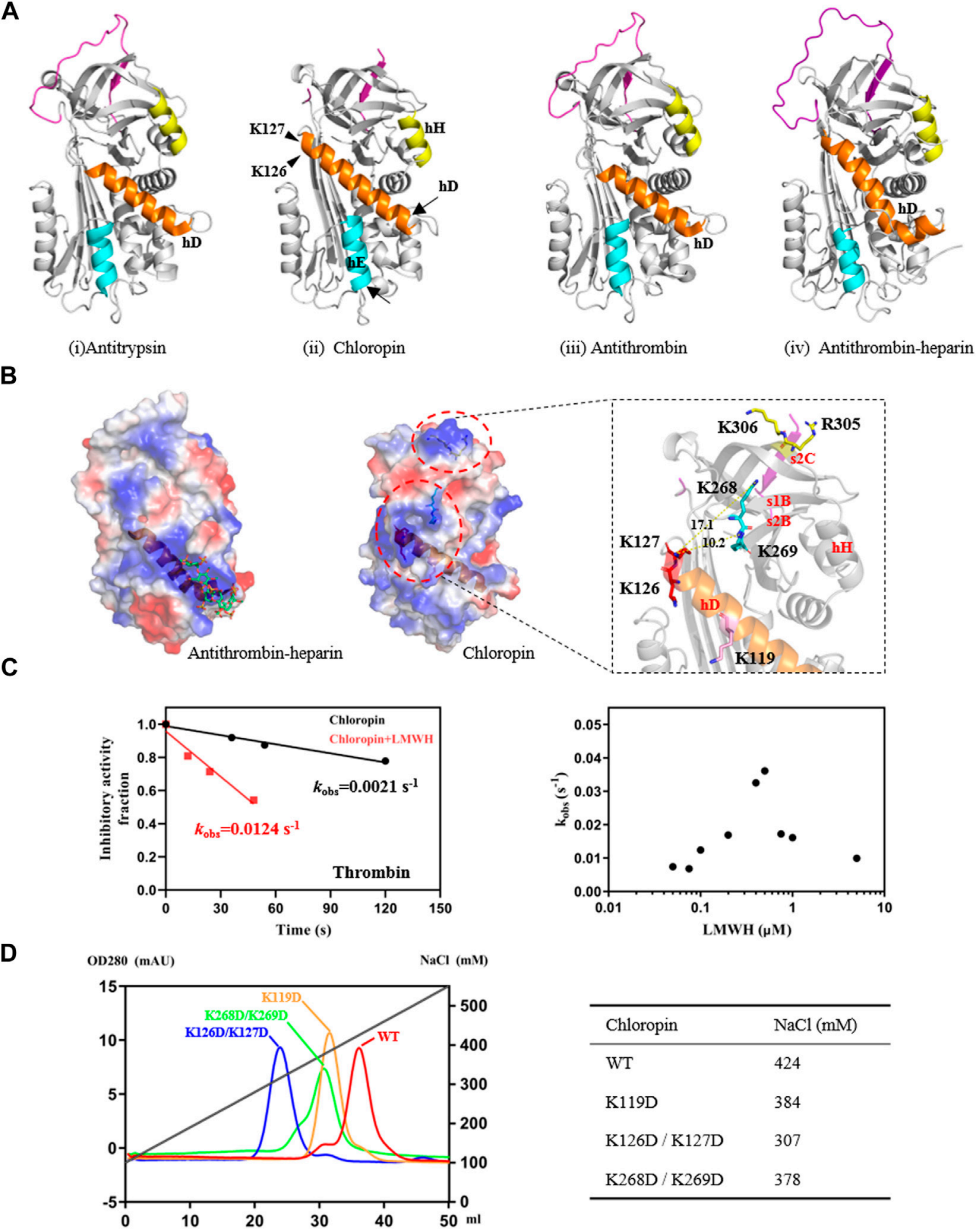
Identification of heparin-binding sites on chloropin. **(A)** Chloropin has similar structural configurations as classic serpin such as antitrypsin (i, PDB 1QLP), antithrombin (iii, PDB 1ATH), and heparin-activated antithrombin (iv, PDB1AZX). Nevertheless, chloropin appeared to have a longer helix D with subtle shifts in the positions of helix E and H (ii). The lower part of helix D of antithrombin plays a key role during heparin activation of antithrombin with helix extensions at both ends (iii, iv). **(B)** Comparison of the electrostatic surfaces of heparin-activated antithrombin and chloropin. Heparin binds a positively charged patch near the lower part of helix D in antithrombin (left). In contrast, there is a positively charged surface area near the top of helix D of chloropin involving residues K119, K126/K127, and K268/K269. Notably, R305 and K306 on top of chloropin are likely involved in the formation of the initial serpin-protease Michaelis complex as mutation of these two residues significantly reduced the reaction between thrombin and chloropin (data not shown). **(C)** Heparin stimulated thrombin inhibition by chloropin. Chloropin (0.4 μM) in the presence or absence of 0.1 μM heparin was incubated with 0.1 μM thrombin for different time intervals with the remaining protease activities plotted to obtain the *k*_obs_ values. There was a ∼6-fold increase in the *k*_obs_ in the presence of 0.1 μM heparin (left) and the acceleration is heparin dose-dependent following a bell-shaped curve (right). **(D)** Heparin-binding affinities of chloropin variants were assessed by a Hitrap heparin column with the salt concentrations in peaks identified.

It is known that eukaryotic serpins are often subject to regulations by their cofactors, such as heparin or heparan sulfate, which has been developed as anticoagulants to prevent blood clots (Gettins, 2002; Gettins and Olson, 2009). A structural comparison of chloropin with antitrypsin, antithrombin and the heparin-activated antithrombin showed that chloropin has the longest helix D (Figure 2A). We then compared the electrostatic surfaces of chloropin with those of other heparin binding serpins such as antithrombin, heparin cofactor II, lamprey angiotensinogen and protein C inhibitor (Figure 2B, Supplementary Figure S2). This showed that chloropin has a relatively smaller positively charged surface area involving K119, K126, K127, K268, and K269 close to the top of helix D while heparin mainly binds to a large positively charged surface area near residue K114 at the bottom of helix D of antithrombin (Jin et al., 1997). To explore whether this positively charged surface of chloropin is related to the regulation by heparin, we mixed different amounts of low molecular weight heparin with chloropin and then assessed its inhibitory activity towards thrombin, which is the most well-studied heparin-binding serine protease. The results showed (Figure 2C) that heparin at 0.1 μM increased the rate of inhibition (*k*_obs_) of thrombin by 5.9-fold and the inhibition followed a bell-shaped heparin dose-dependent curve, a common feature of the so-called bridging mechanism or template mechanism of heparin in promoting protein interaction. The maximum acceleration was observed at 0.5 μM heparin where the rate of inhibition (*k*_obs_) was increased by about 17.2-fold. This showed that the activity of prokaryotic serpins could be similarly modulated by cofactors as seen with eukaryotic serpins where heparin could accelerate serpins’ protease inhibition by a few folds to more than a thousand folds as seen in heparin cofactor II, PAI-1, PCI, antithrombin, vaspin etc (Mottonen et al., 1992; Ulbricht et al., 2017).

To further clarify the binding site of heparin on chloropin, we mutated several positively charged amino acids on the chloropin surface. These charge-reversal mutants, including K119D, K126D/K127D, and K268D/K269D (Figure 2B), were prepared and then firstly assessed for thrombin inhibition by SDS-PAGE. All the mutants could form SDS-stable covalent complexes with thrombin as shown in Supplementary Figure S3. This indicated that the replacement of these surface-exposed lysine or arginine residues into aspartic acids did not affect the thrombin inhibition by chloropin. However, the heparin-mediated acceleration in thrombin inhibition varied significantly amongst these chloropin variants (Table 2). Heparin had a similar accelerating effect on the thrombin inhibition by chloropin K119D mutant like that of the wild-type form, indicating K119 is not involved in heparin-mediated thrombin inhibition. In contrast, heparin could not effectively increase the reaction rates of K126D/K127D and K268D/K269D variants. This suggested that these residues are likely involved in heparin binding for accelerated thrombin inhibition by chloropin (Figure 2D). Heparin could bind across the positively charged surface area of chloropin around residues K126, K127, K268, and K269 to bridge chloropin and thrombin together and promote their interaction (Figure 2B). Therefore, the cofactor binding site of chloropin is distinctly different to those of antithrombin, PCI, kallistatin or vaspin (Jin et al., 1997; Li et al., 2007; Ulbricht et al., 2017; Ma et al., 2020).

**TABLE 2:** Effect of heparin and DNA on the thrombin inhibition by chloropin variants. Chloropin variants (1 μM), thrombin (0.1 μM) and heparin (0.1 μM) were mixed at 20°C for different time intervals and the remaining protease activities were measured with the *k*_obs_ values calculated. The DNA effect was similarly assessed with 375 nM chloropin, 75 nM thrombin and 0.8 nM linear plasmid DNA (lpDNA). Chloropin can be substituted by antithrombin, and linear plasmid DNA can be substituted by Supercoiled plasmid DNA (spDNA).

	*k*_obs_ (×10^−3^ s^−1^) (1 μM chloropin)		*k*_obs_ (×10^−3^ s^−1^) (0.375 μM chloropin)			
	- heparin	+ heparin	- lpDNA	+ lpDNA	- spDNA	+ spDNA
Chloropin WT	8.3 ± 0.3	66.7 ± 0.1	3.5 ± 0.2	14.2 ± 0.2	3.5 ± 0.2	19.3 ± 0.3
Chloropin K119D	9.5 ± 0.2	55.5 ± 0.1	3.8 ± 0.3	5.5 ± 0.4	-	-
Chloropin K126D/K127D	8.6 ± 0.2	10.7 ± 0.2	3.6 ± 0.3	3.5 ± 0.3	-	-
Chloropin K268D/K269D	8.3 ± 1.1	9.6 ± 0.7	3.5 ± 0.2	4.1 ± 0.2	-	-
Antithrombin	-	-	2.7 ± 0.1	3.2 ± 0.2	-	-

### 3.4 DNA accelerates the thrombin inhibition by chloropin

**FIGURE 3:**
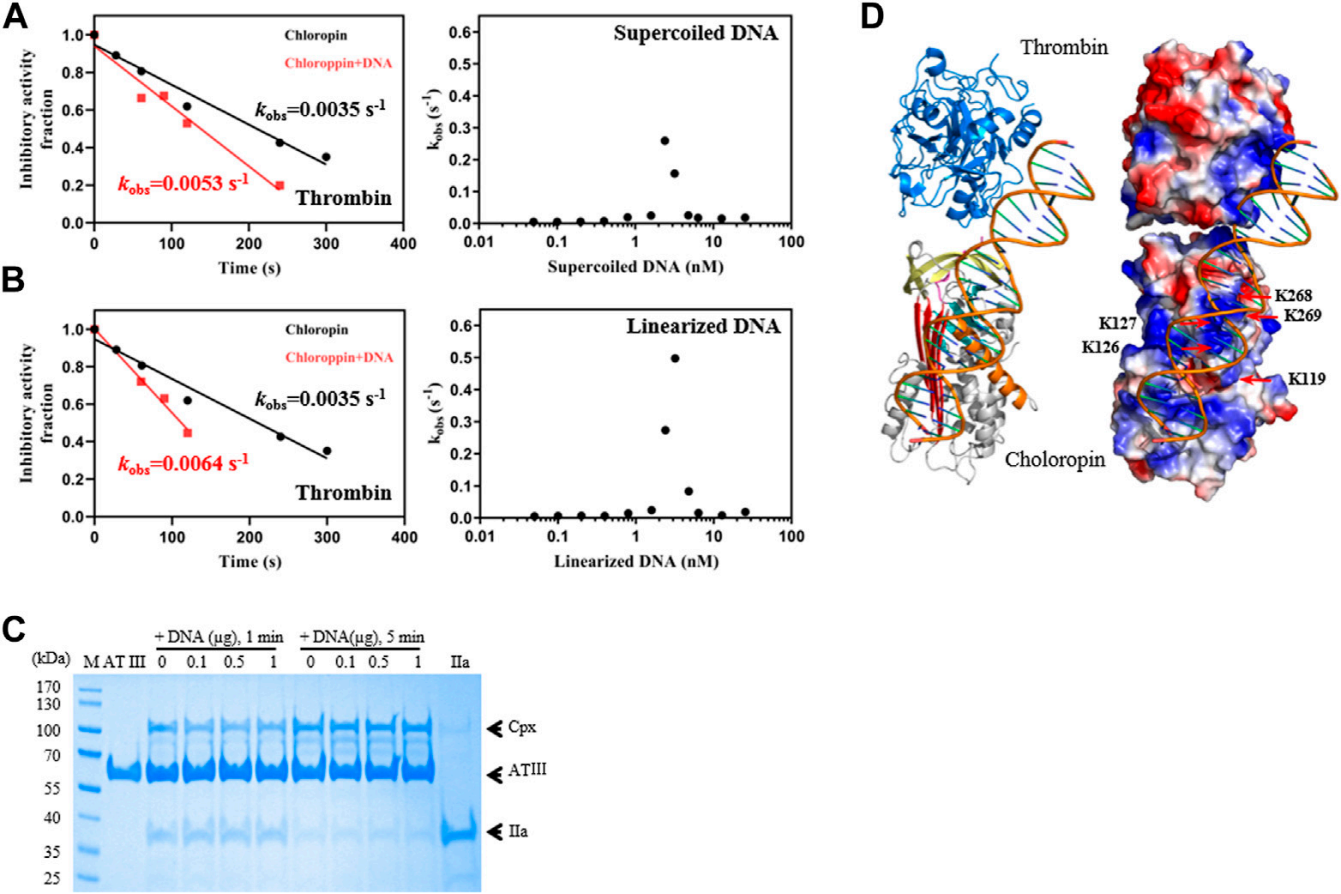
DNA promoted the inhibition of thrombin by chloropin. **(A)** Supercoiled plasmid DNA (0.1 nM) accelerated thrombin inhibition (left) and the acceleration was DNA concentration-dependent and followed a bell-shaped curved (right). Chloropin (375 nM) in the presence or absence of 0.1 nM DNA was incubated with 75 nM thrombin for different time intervals with the remaining protease activity plotted to obtain *k*_obs_. The *k*_obs_ values at different concentrations of DNA (0.05, 0.1, 0.2, 0.4, 0.8, 1.6, 2.4, 3.2, 4.8, 6.4, 12.8, 25.6 nM) were plotted as a scatter chart (right). **(B)** Similar acceleration was also observed with linear plasmid DNA. **(C)** The effect of DNA on the thrombin inhibition by antithrombin. Different amounts of DNA (0, 0.1, 0.5, 1 µg) were incubated with 2 µg antithrombin and 0.5 µg thrombin for 1 min and 5 min respectively, and the samples were then analyzed by SDS-PAGE. Cpx, antithrombin-thrombin covalent complexes; IIa, thrombin. **(D)** A cartoon of the chloropin-thrombin-DNA ternary complex showing the relative positions of thrombin and chloropin. DNA is expected to bind the positively charged surface areas of thrombin and chloropin simultaneously and promote thrombin inhibition.

Unlike eukaryotic serpins, most prokaryotic serpins may never encounter heparin or heparan sulfate in the natural environment. Since heparin and heparan sulfate is composed of linear acidic polysaccharide chains, their negatively charged surface may resemble that of DNA which is also highly negatively charged due to the phosphate group of the nucleotide. Nevertheless, the spatial distribution of the negative charges of DNA is different from that of heparin or heparan sulfate. We first tested the inhibition of thrombin by chloropin in the presence of 0.1 nM of supercoiled plasmid DNA. This showed about a 1.5-fold increase in the *k*_obs_ of chloropin and this acceleration is DNA dose-dependent. The maximum acceleration was obtained between 1.6 and 3.2 nM of supercoiled DNA, where the rate of thrombin inhibition by chloropin was increased by ∼74-fold. The *k*_obs_ gradually decreased at higher DNA concentrations, which yielded a similar bell-shaped dose-dependent curve resembling that of heparin (Figures 3A, B). We also found that linear DNA could promote thrombin inhibition by chloropin even further. The maximum acceleration was obtained around 2.4–4.8 nM of linear DNA with the rate of inhibition increased by 142.2-fold. Thus, DNA is more efficient in promoting the interaction between chloropin and thrombin than heparin. The different effect of supercoiled and linear DNA likely arises from their differences in the overall shape and charge distribution. Notably, DNA had a very limited acceleration effect on the thrombin inhibition by all the chloropin variants (K119D, K126D/K127D, K268D/K269D) at the tested concentration (Table 2). Therefore, all these residues seemed to be involved in the DNA accelerated thrombin inhibition by chloropin. Furthermore, we have also tested the length effect of DNA oligos on thrombin inhibition by chloropin and found that oligoes shorter than 40 bp are inefficient in promoting thrombin inhibition (Supplementary Table S1). Therefore, it requires oligos of ∼40 bp to bridge thrombin and chloropin together.

We also tested if DNA could similarly accelerate the thrombin inhibition by antithrombin. However, little acceleration could be detected from the activity assay (Table 2), we further assessed this by SDS-PAGE analysis where antithrombin was mixed with thrombin in the presence of different amounts of DNA for 1 min or 5 min respectively. As shown in Figure 3C, antithrombin formed covalent antithrombin-thrombin complexes readily in the absence of DNA, however, there is little increase in the formation of complexes with the addition of DNA. Therefore, the effect of DNA on accelerated thrombin inhibition by chloropin is somewhat specific. This difference between chloropin and antithrombin likely lies in the unique surface charge distribution of chloropin which could favorably interact with DNA and allow DNA to bridge chloropin and thrombin efficiently.

Subsequently, a molecular model of chloropin-thrombin-DNA ternary complex was generated to assess the relative positions of the components. This showed that it is feasible for DNA to bind across the helix D of chloropin and then engage with the positively charged surface area of thrombin (Figure 3D) serving as a template to promote protease inhibition. As DNA is present in the cytosol of bacteria and many prokaryotic serpins are located intracellularly, we speculate that DNA may be a natural modulator of chloropin in nature.

## 4 Discussion

The activities of eukaryotic inhibitory serpins are often modulated by their physiological cofactors or ligands. For example, heparin or heparan sulfate anchored on the endothelium surface could recruit several serpins such as antithrombin (Jin et al., 1997), heparin cofactor II (Baglin et al., 2002), PAI-1 (Ehrlich et al., 1991; Podor et al., 2000), protease nexin 1 (Li and Huntington, 2012), protein C inhibitor (Li et al., 2008), vaspin (Ulbricht et al., 2018), etc. and accelerate the inhibition of thrombin in a local microenvironment. The detailed heparin binding on these inhibitory serpins has been well characterized with the binding sites often located to positively charged surface areas near helix D or H of the serpins. As heparin also binds thrombin, it could promote the serpin-thrombin interaction through a template or bridging mechanism where one heparin chain binds serpin and protease simultaneously and accelerates the rate of inhibition. Since very little is known about the modulation of prokaryotic serpins, here we have prepared a bacteria serpin, chloropin, and characterized the cofactor effect on its protease inhibition.

Our crystallographic study showed that chloropin has a canonic inhibitory serpin conformation with a surface-exposed reactive loop. Activity assay indicated that chloropin could inhibit serine proteases such as thrombin and KLK7 with second-order rate constants 4.5×10^4^ M^−1^s^−1^ and 2.5×10^4^ M^−1^s^−1^, respectively, in line with other prokaryotic serpins. For example, thermopin inhibited chymotrypsin with a rate of 8.4×10^4^ M^−1^s^−1^ (Irving et al., 2003). Prokaryotic serpin from Bifidobacterium inhibited neutrophil elastase with a rate of 4.7×10^4^ M^−1^s^−1^ (Ivanov et al., 2006). Aeropin inhibited chymotrypsin at a rate of 1.8×10^5^ M^−1^s^−1^ (Cabrita et al., 2007). Nevertheless, these inhibition rates are slightly slower than the rates of eukaryotic inhibitory serpins which are often in the range of 10^5^–10^7^ M^−1^s^−1^. We have found that heparin could accelerate chloropin’s inhibition of thrombin by more than 17-fold with heparin binding to a positively charged surface area near the top of helix D of chloropin. Notably, heparin-binding of chloropin does not induce any significant conformational changes of the serpin when assessed by fluorescent titrations (data not shown). As heparin promoted chloropin’s thrombin inhibition in a bell-shaped dose-dependent curve, this suggested that heparin mediated the acceleration through the common template mechanism.

Since heparin and heparan sulfate are mainly found in higher organisms, we reckon it is highly unlikely that chloropin would encounter heparin and heparan sulfate in nature. We reasoned that other linear charged molecules such as DNA may have a similar effect on the interaction between chloropin and thrombin. Previously DNA was shown to bind α_1_-antichymotrypsin without affecting its inhibitory ability (Naidoo et al., 1995). It has been shown that DNA could promote the inhibition of cathepsin V by a nuclear serpin MENT where DNA could bind to the unique M-loop of MENT (Ong et al., 2007). Here, we tested the effect of DNA on the thrombin inhibition by chloropin and showed that supercoil DNA could increase the rate of thrombin inhibition by 74-fold while linear DNA could increase the rate by 142-fold following a similar bell-shaped dose-dependent curve. This makes chloropin one of the fastest prokaryotic serpins with a second order rate constant of 3.6×10^6^ M^−1^s^−1^. Thus, DNA is far more efficient than heparin in promoting thrombin inhibition by chloropin. In contrast, DNA could not accelerate the antithrombin’s inhibition of thrombin (Figure 3C). This specificity of DNA towards chloropin likely arises from the spatial distribution of phosphate groups of DNA and the positively charged residues on the surface of chloropin. Notably, sequence analysis of chloropin indicated that chloropin has a conserved signal peptide for extracellular secretion. This indicated that chloropin is secreted by bacterial to inhibit proteases in the extracellular environment where DNA might also be present due to burst bacterial or similar secretion by bacterial (Costa et al., 2015; Green and Mecsas, 2016).

However, it remains elusive on the targeting proteases of chloropin and many other prokaryotic serpins (Kang et al., 2006; Turroni et al., 2010; Cuiv et al., 2013; Akermi et al., 2019). Little is known if these bacteria would encode any endogenous proteases or if they would encounter any environmental proteases. It has been suggested that a serpin encoded by gut bacterium *Bifidobacterium longum* may encounter both pancreatic elastase and neutrophil elastase in human gastrointestinal tract and provide protection against exogenous proteolysis (Ivanov et al., 2006). Nevertheless, our findings here indicate that chloropin would be most efficient in inhibiting a thrombin-like serine protease. As mammalian serpins also have other non-inhibitory functions such as molecular chaperones, hormone carriers etc., it remains unknown if prokaryotic serpins could have similar functions. Clearly further research on prokaryotic serpins is required to address these questions.

Our study here also sheds light on the origin of the prokaryotic serpins. It was previously proposed that prokaryotic serpins were acquired *via* a rare interkingdom horizontal gene transfer event (Irving et al., 2002), however, a recent comprehensive phylogeny analysis of serpins from all kingdoms of life has found no evidence of horizontal gene transfer (Spence et al., 2021) and showed, in instead, that the common ancestry of prokaryotic serpins may descend from a single common ancestor distinctly different from the last common ancestor of the chordate serpins (Spence et al., 2021). Prokaryotic serpins likely evolved independent of the eukaryotic serpins but retained the key serpin inhibition mechanism. Our studies are consistent with this notion with chloropin inhibiting target proteases through the same suicidal-inhibition mechanism. More importantly, chloropin has evolved to utilize a different set of residues on serpin surface for specific DNA binding and activity modulation. This unique modulation feature of chloropin is unlikely acquired through horizontal gene transfer from eukaryotic serpins.

In conclusion, our studies here have identified chloropin as the first prokaryotic serpins to be regulated by DNA binding and suggest that DNA may be a natural cofactor or modulator of chloropin in protecting bacterial cells from either endogenous or exogenous environmental thrombin-like proteases.

## Data Availability

The datasets presented in this study can be found in online repositories. The names of the repository/repositories and accession number(s) can be found in the article/Supplementary Material.
