# Supplementary material for: DNA accelerates the protease inhibition of a bacterial serpin chloropin
Source: Front Mol Biosci. 2023 Mar 29;10:1157186. doi: 10.3389/fmolb.2023.1157186 (PMC10090351; doi:10.3389/fmolb.2023.1157186)
Supplement: Supplementary file 1 [file DataSheet2.docx]

Supplementary Material

DNA accelerates the protease inhibition of a bacterial serpin chloropin

Jiawei Xu^†^, Wei Ye^†^, Ting Ting Yang, Teng Yan, Haiyan Cai^*^, Aiwu Zhou^*^, Yufeng Yang^*^

*** Correspondence:** Haiyan Cai: hycai@shsmu.edu.cn; Aiwu Zhou: awz20@shsmu.edu.cn; Yufeng Yang: yfyang@zmu.edu.cn

**^†^ These authors contributed equally to this work.**

# Supplementary Tables

Supplementary Table 1. The effect of different lengths of DNA oligos on the inhibition rate of chloropin. Chloropin (25 nM) was mixed with DNA (25 nM) oligos of different lengths and then incubated with 5 nM thrombin for different time intervals and the remaining protease activity was plotted to obtain *k*_obs_ values.

^a^ blank control.

| linear DNA | *k*_obs_ (×10^-4^ s^-1^) |
| --- | --- |
| -^a^ | 2.1 ± 0.1 |
| 10 bp | 2.3 ± 0.2 |
| 15 bp | 2.4 ± 0.2 |
| 20 bp | 2.3 ± 0.2 |
| 40 bp | 5.8 ± 0.2 |
| 60 bp | 4.0 ± 0.1 |
| 80 bp | 2.8 ± 0.1 |

# Supplementary Figures


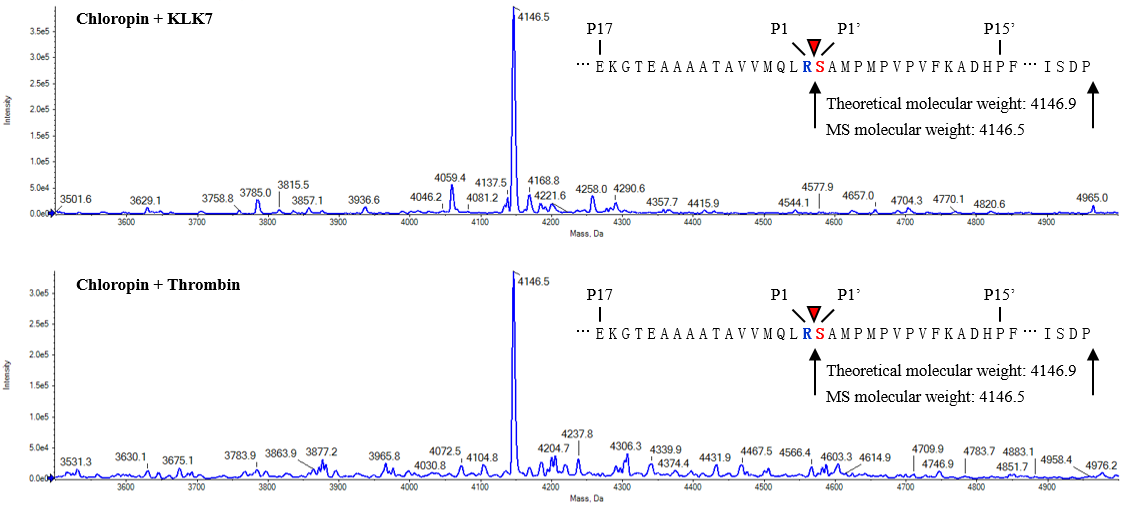


**Supplementary Figure 1.** Mass spectrometry analysis of protease cleavage site in the reactive center loop of chloropin. Chloropin was mixed with proteases KLK7 or thrombin and then subject to mass spectrometry analysis. The C-terminal peptide released by KLK7 (top) and thrombin (bottom) has an almost identical mass of 4146.5 Da, which corresponds to a cleavage site between Arg383 and Ser384.


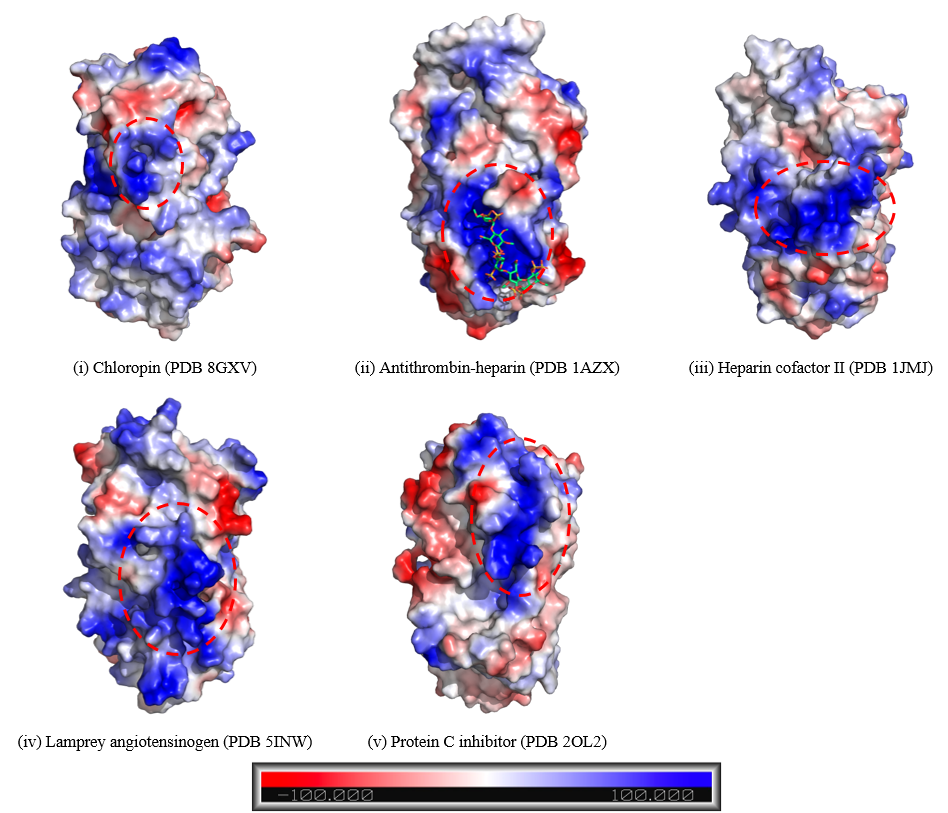


**Supplementary Figure 2.** The electrostatic surfaces of heparin binding serpins (chloropin, antithrombin-heparin, heparin cofactor II, lamprey angiotensinogen and protein C inhibitor). The positive charges are in blue and negative charges are in red. The heparin binding patches are highlighted in dashed oval lines.


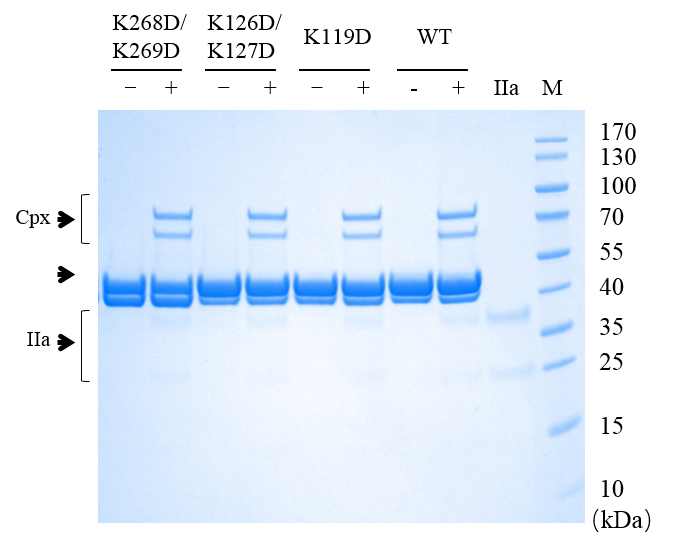


**Supplementary Figure 3.** Assessment of thrombin inhibition by chloropin variants on SDS-PAGE. Chloropin variants (6 μg) including the wild type, K119D, K126D/K127D, and K268D/K269D, were incubated with thrombin (0.5 μg) respectively at 37°C for 20 min and the samples were then analyzed by reducing SDS-PAGE.


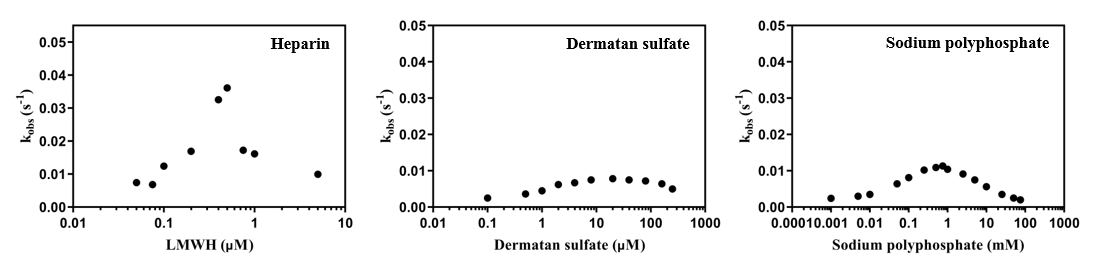


**Supplementary Figure 4.** Stimulation effect of heparin (left), dermatan sulfate (middle) and sodium polyphosphate (right) on thrombin inhibition by chloropin. In the presence of different concentrations of heparin or dermatan sulfate or sodium polyphosphate, chloropin (0.4 μM) was incubated with 0.1 μM of thrombin for different time intervals and the remaining protease activity was plotted to obtain *k*_obs_ values. The acceleration follows a dose-dependent bell-shaped curve, with the maximum enhancement of 17.2-fold for heparin, 3.7-fold for dermatan sulfate and 5.4-fold for sodium polyphosphate.
